# Impact of perfusion method on perioperative red blood cell transfusions and new-onset postoperative atrial fibrillation in mitral valve surgery patients

**DOI:** 10.1177/02676591221122351

**Published:** 2022-08-23

**Authors:** Jenni Räsänen, Sten Ellam, Juha Hartikainen, Auni Juutilainen, Jari Halonen

**Affiliations:** 1 Institute of Clinical Medicine, School of Medicine, 220881 University of Eastern Finland, Kuopio, Finland; 2 Heart Center, 60650 Kuopio University Hospital, Kuopio, Finland; 3 Department of Anesthesiology and Operative Services, 60650 Kuopio University Hospital, Kuopio, Finland

**Keywords:** cardiac surgery, cardiopulmonary bypass, conventional extracorporeal circulation, minimal invasive extracorporeal circulation, mitral valve surgery, new-onset atrial fibrillation, postoperative atrial fibrillation, red blood cell transfusion

## Abstract

**Introduction:**

Red blood cell (RBC) transfusions are common in cardiac surgery and reportedly associated with increased mortality and morbidity, including increased risk of postoperative new-onset atrial fibrillation (NOAF). The aim of this study was to compare minimal invasive extracorporeal circulation (MiECC) and conventional extracorporeal circulation (CECC) in terms of RBC transfusions and the incidence of NOAF in mitral valve surgery.

**Methods:**

The study population consisted of 89 MiECC and 169 CECC patients undergoing mitral valve surgery as an isolated procedure (80.6% of the patients) or in combination with coronary artery bypass grafting (19.4% of patients). 79.4% of the patients were male and the mean age was 62.1 years.

**Results:**

30.0% of patients aged < 65 years and 48.1% of patients aged ≥ 65 years needed RBC transfusion. The overall need for RBC transfusions did not differ between the treatment groups. Among patients < 65 years of age transfusions of ≥ 3 units were less frequent in MiECC than in CECC patients (OR 0.31, 95% CI 0.10–0.98, *p* = 0.045). The overall incidence of NOAF was 41.8% with no significant difference between MiECC and CECC groups. Red blood cell transfusions were associated with an increased risk of NOAF in an unadjusted analysis but not after adjustment for age and sex (OR 1.25, 95% CI 0.64–2.43, *p* = 0.515).

**Conclusions:**

In mitral valve surgery MiECC compared to CECC was associated with less need of RBC units and platelets, particularly in patients aged < 65 years. Use of RBC transfusions was associated with increased risk of NOAF significantly only in unadjusted analysis.

## Introduction

The use of allogeneic blood products is common in cardiac surgery. The need of red blood cells (RBC), the most frequently used allogeneic blood products, is related to the invasiveness of the surgical procedure, the use of anticoagulation and antithrombotic agents as well as the use of cardiopulmonary bypass. Red blood cell transfusions have been shown to negatively impact patient recovery, morbidity, and mortality.^1,2^ It is of special importance to reduce the use of RBC transfusions in cardiac surgery by maintaining perioperative hemostasis and minimizing postoperative bleeding. Therefore, in 2017, European Association for Cardio-Thoracic Surgery (EACTS) and the European Association of Cardiothoracic Anaesthesiology (EACTA) published guidelines on patient blood management to provide clinicians with practical and evidence-based recommendations.^3^

There is evidence suggesting that RBC transfusions increase the risk of new-onset atrial fibrillation (NOAF) after cardiac surgery.^4,5^ NOAF, i.e. new-onset atrial fibrillation in patients without a preoperative history of atrial fibrillation, is the most common arrhythmia after cardiac surgery. The incidence of NOAF after isolated coronary artery bypass surgery (CABG) ranges from 23% to 36%, increasing up to 42% in patients undergoing mitral valve surgery, and up to 60% in those scheduled for combined CABG and mitral valve procedure.^5–10^

The pathogenesis of NOAF is not fully understood. Systemic inflammation and oxidative stress have been associated with the development of NOAF.^11–15^ These conditions are considered to be related to the use of cardiopulmonary bypass and reperfusion injury after cardioplegic arrest.^16–18^ A position paper from the Minimal invasive Extra-Corporeal Technologies international Society (MiECTiS) acknowledges that the minimal invasive extracorporeal circulation (MiECC) might be useful in reducing systemic inflammation and oxidative stress and the incidence of NOAF associated with cardiac surgery.^19^

The aim of this study was to compare MiECC and conventional extracorporeal circulation (CECC) in terms of RBC transfusions and the incidence of NOAF, and to settle whether RBC transfusions are associated with the risk of NOAF in mitral valve surgery.

## Methods

### Study design and patient population

The data was collected from Kuopio University Hospital cardiac surgery database. The study included 281 consequent patients scheduled for mitral valvuloplasty or mitral valve replacement from January 2013 to December 2016. In combination to mitral valve procedure 50 patients (19.4%) underwent CABG, but other combined procedures were excluded. 23 patients were excluded because of missing data on RBC transfusions. The final study population consisted of 258 patients, of whom 89 underwent perfusion with MiECC and 169 with CECC.

### Minimal invasive extracorporeal circulation

The type III MiECC system set contains Quadrox-i adult microporous membrane oxygenator with integrated arterial filter, venous bubble trap (VBT), tubing and reservoir with a possibility to give blood back to the venous line. All elements of the system had the Softline coating (Maquet). The type III system was built around Cardiohelp console (Maquet, Rastatt, Germany) which included centrifugal pump, online monitoring of temperature, hemoglobin, hematocrit, and venous oxygen saturation. The circuit prime consisted of 1000 mL of Ringer-Acetate solution (Baxter Oy, Helsinki, Finland) and 10,000 IU heparin (Leo, Ballerup, Denmark). Air removal was possible from venting system, VBT and arterial filter and both venous and arterial sides had bubble alarms and there was a level sensor on VBT. Venting was performed from the aortic root and/or from pulmonary artery via a drip chamber to the venous line driven by negative pressure of the venous side. Calafiore-type warm blood cardioplegia was used at 34°C driven by arterial line positive pressure without separate pump. Standalone flow/volume meter (Sono TT; Emtec GmbH, Finning, Germany) monitored cardioplegia flow and volume on the cardioplegia line. Initial antegrade dose and repeated boluses after 15–25 min, depending on the type and phase of procedure, antegrade, retrograde or coronary ostial routes were used.

### Conventional extracorporeal circulation

S3 or S5 heart-lung machines with roller pumps (LivaNova, Mirandola, Italy) were used. There were two different customized perfusion sets used on perfusion. One set was from Maquet including open hard-shell venous reservoir, Quadrox-i adult microporous membrane oxygenator with integrated arterial filter and tubing, with all elements of circuit Softline-coated. The other set was LivaNova including open hard-shell venous reservoir, membrane oxygenator and tubing, with all parts of circuit phosphorylcholine-coated. Circuit prime consisted of 2000 mL Ringer-Acetate solution, 3.0 *g* of tranexamic acid and 10,000 IU of heparin. Buckberg-type tepid blood cardioplegia (4:1 and 8:1 ratios) was administered at 23°C via Plegiox heat exchanger (Maquet) and separate double-head roller pump. Initial antegrade dose and repeated boluses after 15–25 min, depending on type and phase of procedure, antegrade, retrograde or coronary ostial routes were used. The same suction system was used in both CECC methods. Temperature of patients was cooled to 34°C and rewarmed to 36°C before cannulation.

3.0 *g* of tranexamic acid was given to all patients intravenously and in CECC group another 3.0 *g* of tranexamic acid was added to perfusion set priming solution. Hepcon (Medtronic, Minneapolis, MN, USA) protocol was used. The activated coagulation time target was 480 s in both perfusion groups. Heparin concentration was controlled at 20–30 min and accurate heparin concentration was maintained during the perfusion. Heparin was reserved with protamine according to Hepcon calculation at the end of the procedure. If the patient was treated with warfarin, INR > 1.6 was the trigger for fresh frozen plasma infusion. Patients were transferred to intensive care unit for at least overnight after the operation.

### Outcomes

The primary outcome of this study was the use of RBC transfusions. The patients were categorized in four groups according to the number of RBC units transfused (0, 1–2, 3–6 and > 6 RBC units). In addition, patients were categorized by age of < 65 years and ≥ 65 years. The trigger for RBC transfusion was a hemoglobin level below 80 *g*/L during peri- and postoperative periods. The secondary outcome of the study was the incidence of NOAF episode > 5 min during the postoperative stay in a patient without a history of preoperative atrial fibrillation.

### Ethics

The study complied with the Declaration of Helsinki. It was approved by the Research Ethics Committee of the Northern Savo Hospital District (No. 1694/13.02.00/2019). Informed consent was not required because the study was register-based.

### Data analysis and statistical methods

Statistical analysis was performed with IBM SPSS Statistics 25.0 software for Windows (IBM corp. Armonk, NY, USA). Categorical variables were analysed with the Chi-square test or Fisher’s exact test and continuous variables with the Student’s t-test and Mann-Whitney U test when appropriate. Logistic regression analysis with adjustment for age and sex was used to evaluate the association between the use of RBC transfusions and the incidence of NOAF. The data are presented as means and standard deviations or frequencies and percentages when appropriate. A *p*-value < 0.05 was considered statistically significant.

## Results

The preoperative clinical characteristics in MiECC and CECC groups were well balanced with no statistically significant differences (Table 1). In general, patients aged ≥ 65 years had more often a history of comorbidities than their younger peers. In particular, the history of preoperative atrial fibrillation (*p* < 0.001), previous stroke or transient ischemic attack (*p* = 0.021), lower eGFR (< 0.001) and diabetes (*p* = 0.002) were more frequent in patients aged ≥ 65 years. Also, the combined procedure (mitral valve procedure + CABG) was more frequent in patients aged ≥ 65 years (*p* < 0.001).

**Table 1.** Preoperative clinical characteristics.

	MiECC *n* = 89		CECC *n* = 169		All *n* = 258	p-value		
	< 65 years (*n* = 50)	≥ 65 years (*n* = 39)	< 65 years (*n* = 100)	≥ 65 years (*n* = 69)		< 65years MiECC versus CECC	≥ 65 years MiECC versus CECC	All MiECC versus CECC
Sex, male	44 (88.0%)	23 (59.0%)	87 (87.0%)	50 (72.5%)	204 (79.1%)	0.862	0.150	0.278
Age, years	54.1 (8.1)	72.6 (5.6)	54.0 (7.2)	73.9 (5.2)	62.1 (11.6)	0.926	0.213	0.959
BMI, kg/m^2^	26.6 (4.7)	25.9 (3.5)	25.8 (4.3)	25.8 (3.7)	26.0 (4.1)	0.290	0.859	0.343
eGFR, ml/min/1.73 m^2^	93.1 (18.2)	75.4 (15.8)	89.5 (15.1)	70.4 (17.6)	83.0 (18.9)	0.208	0.142	0.143
Hemoglobin, g/l	140.2 (16.2)	136.9 (17.0)	140.5 (19.6)	137.6 (16.6)	139.1 (17.8)	0.938	0.826	0.814
Smoking (current or previous)	17 (34.7%)	27 (27.8%)	9 (25.0%)	17 (26.2%)	70 (28.3%)	0.394	0.899	0.570
LVEF, %	60.4 (9.9)	59.4 (10.9)	61.5 (11.1)	61.3 (10.5)	61.0 (10.6)	0.557	0.368	0.294
History of atrial fibrillation	4 (8.0%	16 (41.0%)	12 (12.0%)	18 (26.1%)	42 (16.3%)	0.454	0.108	0.362
Stroke or TIA	2 (4.0%)	3 (7.7%)	2 (2.0%)	7 (10.1%)	14 (5.4%)	0.474	0.673	0.921
ASO	1 (2.0%)	2 (5.1%)	1 (1.0%)	2 (2.9%)	6 (2.3%)	0.615	0.556	0.419
Diabetes	4 (8.0%)	6 (15.4%)	4 (4.0%)	13 (18.8%)	27 (10.5%)	0.304	0.650	0.769
Kidney disease	1 (2.0%)	1 (2.6%)	2 (2.0%)	6 (8.7%)	10 (3.9%)	1.000	0.214	0.325
COPD	2 (4.0%)	3 (7.7%)	2 (2.0%)	3 (4.3%)	10 (3.9%)	0.474	0.466	0.293
UAP	1 (2.0%)	2 (5.1%)	3 (3.0%)	4 (5.8%)	10 (3.9%)	0.720	0.884	0.760
NYHA						0.465	0.417	0.428
No symptoms	7 (14.0%)	2 (5.1%)	8 (8.0%)	0 (0.0%)	17 (6.6%)			
1	7 (14.0%)	3 (7.7%)	20 (20.0%)	5 (7.2%)	35 (13.6%)			
2	23 (46.0%)	11 (28.2%)	39 (39.0%)	24 (34.8%)	97 (37.6%)			
3	9 (18.0%)	15 (38.5%)	27 (27.0%)	27 (39.1%)	78 (30.2%)			
4	4 (8.0%)	8 (20.5%)	6 (6.0%)	13 (18.8%)	31 (12.0%)			
Warfarin	1 (2.0%)	3 (7.7%)	5 (5.0%)	14 (20.3%)	23 (8.9%)	0.084	0.071	0.377
Clopidogrel	1 (2.0%)	0 (0.0%)	0 (0.0%)	0 (0.0%)	1 (0.4%)	-	0.167	0.156
LMWH	1 (2.0%)	1 (2.6%)	9 (9.0%)	4 (5.8%)	15 (5.8%)	0.442	0.076	0.105
Combined with CABG	4 (8%)	10 (26%)	14 (14%)	22 (32%)	50 (19.4%)	0.495	0.286	0.282

ASO, arteriosclerosis obliterans; BMI, body mass index; CABG, coronary artery bypass grafting; CECC, conventional extracorporeal circulation; COPD, chronic obstructive pulmonary disease; eGFR, estimated glomerular filtration rate; LMWH, low molecular weight heparin; LVEF, left ventricular ejection fraction; MiECC, minimal invasive extracorporeal circulation; NYHA, New York Heart Association class; TIA, transient ischemic attack; UAP, unstable angina pectoris; The values are n (%) or mean (standard deviation).

A total of 33 patients (37.1%) in MiECC group and 64 (37.9%) in CECC group were given RBC transfusions (*p* = 0.234) (Table 2). The need for platelet transfusions was lower in MiECC group (*p* = 0.022). The need of RBC transfusion was higher in the group of patients aged ≥ 65 years than among the younger patients (*p* = 0.020).

**Table 2.** Peri- and postoperative characteristics according to age group and perfusion methods.

	MiECC			CECC			*p*-value		
	< 65 years (*n* = 50)	≥ 65 years (*n* = 39)	All (*n* = 89)	< 65 years (*n* = 100)	≥ 65 years (*n* = 69)	All (*n* = 169)	< 65 years (*n* = 150) MiECC versus CECC	≥ 65 years (*n* = 108) MiECC versus CECC	All (*n* = 258) MiECC versus CECC
RBC transfusion							0.030	0.698	0.234
0 U	35 (70.0%)	21 (53.8%)	56 (62.9%)	70 (70.0%)	35 (50.7%)	105 (62.1%)			
1–2 U	11 (22.0%)	7 (17.9%)	18 (20.2%)	9 (9.0%)	12 (17.4%)	21 (12.4%)			
3–6 U	4 (8.0%)	7 (17.9%)	11 (12.4%)	13 (13.0%)	18 (26.1%)	31 (18.3%)			
>6 U	0 (0.0%)	4 (10.3%)	4 (4.5%)	8 (8.0%)	4 (5.8%)	12 (7.1%)			
Platelet transfusion*	2 (4.2%)	3 (7.9%)	5 (5.8%)	14 (14.1%)	12 (18.5%)	26 (15.9%)	0.069	0.142	0.022
Aortic cross-clamp time (min)	100 (30)	101 (29)	100 (29)	112 (31)	119 (37)	115 (34)	0.019	0.008	<0.001
Perfusion time (min)	119 (34)	123 (36)	121 (35)	133 (47)	142 (44)	136 (46)	0.062	0.032	0.006

CECC, conventional extracorporeal circulation; MiECC, minimal invasive extracorporeal circulation; *The data on platelet transfusions were missing from three study participants of MiECC group. The values are n (%) or mean (standard deviation).

**Figure 1:**
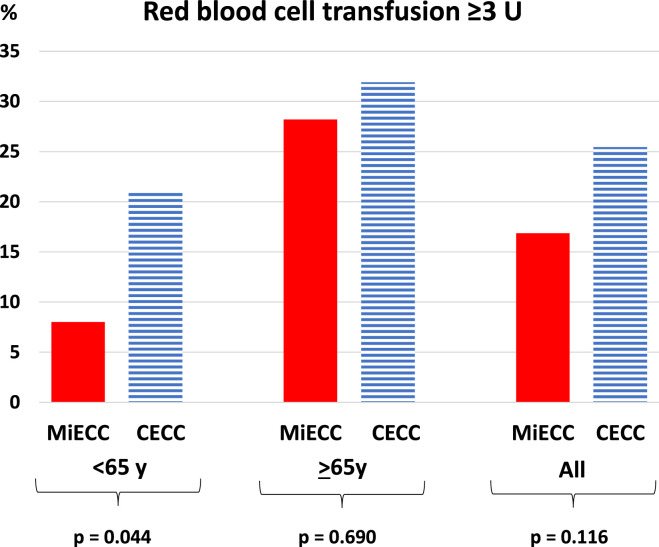
. Red blood cell transfusions ≥ 3 units in mitral valve surgery according to the method of perfusion and age group. *p*-values stand for the difference between MiECC and CECC groups analysed by the Chi-square test.

Among patients under 65 years of age the total amount of RBC units transfused was lower in the MiECC than in the CECC group (*p* = 0.030), although 30% of patients both in MiECC and CECC groups were treated with at least one unit of RBCs. Red blood cell transfusions of 3 units or more were less frequent in MiECC group: In MiECC group 4 (8%) and in CECC group 21 (21%) of the patients received 3 units or more RBC [odds ratio (OR) 0.31, 95% CI 0.10–0.98, *p* = 0.045, (adjusted for age and sex)] (Figure 1). Similarly, in MiECC group there was no need for RBC transfusions of more than 6 units, whereas this was true for 8.0% of patients in the CECC group.

In the group of patients aged ≥ 65 years, 18 (46.2%) of the MiECC patients and 34 (49.3%) of the CECC patients received at least one unit RBC transfusion with no statistically significant difference between the groups (*p* = 0.698). Nor was there a statistically significant difference between MiECC and CECC groups in RBC transfusion of 3 units or more (*p* = 0.690).

Perfusion time and aortic cross-clamp time were significantly lower in MiECC group than in CECC group (*p* = 0.006 and *p* < 0.001, respectively) (Table 2). Corresponding differences were true for cross-clamp time in the groups of patients < 65 years of age (*p* < 0.019) and ≥ 65 years of age (*p* = 0.008).

In a sub-analysis of patients without preoperative AF history, 87 (41.8%) patients developed NOAF (Table 3). The incidence of NOAF was higher in patients aged ≥ 65 years (60.8%) than among younger patients (31.3%) (*p* < 0.001). However, there were no statistically significant differences in the incidence of NOAF between MiECC and CECC groups. In the whole population 42.0% of the MiECC and 41.7% of the CECC patients developed NOAF (*p* = 0.967). Respectively, 30.4% and 31.8% in the group of patients < 65 years of age (*p* = 0.870) and 65.2% and 58.8% in the group of patients ≥ 65 years of age (*p* = 0.602) developed NOAF. In univariate analysis RBC transfusion was associated with an elevated risk of NOAF (OR 1.86, 95% CI 1.05–3.29, *p* = 0.033), but not after adjustment for age and sex (OR 1.25, 95% CI 0.64–2.43, *p* = 0.515).

**Table 3:** . Incidence of new-onset postoperative atrial fibrillation according to age group and perfusion method.

	MiECC			CECC			p-value		
	< 65 years (*n* = 46)	≥ 65 years (*n* = 23)	All (*n* = 69)	< 65 years (*n* = 88)	≥ 65 years (*n* = 51)	All (*n* = 139)	< 65 years MiECC versus CECC	≥ 65 years MiECC versus CECC	All MiECC versus CECC
NOAF	14 (30.4)	15 (65.2)	29 (42.0)	28 (31.8)	30 (58.8)	58 (41.7)	0.870	0.602	0.967

CECC, conventional extracorporeal circulation; MiECC, minimal invasive extracorporeal circulation; NOAF, new-onset postoperative atrial fibrillation. The values are n (%) of patients without preoperative atrial fibrillation. The values are n (%).

The duration of hospital stay was 6.1 days with no statistical difference between MiECC and CECC groups (*p* = 0.766) (Table 4). Finally, the 30-day all-cause mortality between MiECC (1.1%) and CECC groups (0.6%) did not differ (*p* = 0.643).

**Table 4:** . Postoperative clinical characteristics.

	MiECC			CECC			p-value		
	< 65 years (*n* = 50)	≥ 65 years (*n* = 39)	All	< 65 years (*n* = 100)	≥ 65 years (*n* = 69)	All	< 65 years MiECC versus CECC	≥ 65 years MiECC versus CECC	All MiECC versus CECC
30-day mortality	0 (0.0%)	1 (2.6%)	1 (1.1%)	1 (1.0%)	0 (0.0%)	1 (0.6%)	0.478	0.181	0.643
Length of hospital stay (days)	5.3 (2.0)	6.9 (3.1)	6.0 (2.7)	5.7 (2.7)	6.7 (4.2)	6.1 (3.4)	0.333	0.821	0.766

MiECC, minimal invasive extracorporeal circulation; CECC, conventional extracorporeal circulation. The values are n (%) or mean (standard deviation).

## Discussion

A novel finding of this study was that in patients under the age of 65 years undergoing mitral valve surgery MiECC was associated with less use of RBC units and platelets than CECC. There was no difference in the incidence of NOAF or in 30-day mortality between the perfusion methods. In univariate analysis RBC transfusion was associated with increased risk of NOAF in all patients, consistently with previous findings.^4,5^

Evolution of cardiopulmonary bypass methods may help to reduce mortality and morbidity related to cardiac surgery. The MiECC method reduces hemodilution, preserves hematocrit, and reduces postoperative bleeding and the need for RBC transfusions.^19^ In comparison to CECC, it also reduces blood-air and blood-artificial material contacts, which are considered to result in systemic inflammation.^16,20^ Accordingly, lower levels of inflammatory marker interleukin-6 have been reported after MiECC.^21,22^

In our study MiECC treated patients were given less RBC transfusion compared to CECC treated patients. This is in line with previous studies in which MiECC has been associated with reduced need for RBC transfusion in cardiac surgery. However, these studies have mainly focused on cardiac surgery in general or on isolated CABG surgery. In a study in CABG patients, the median number of transfusions of blood products per patient was 0.8 in MiECC group and 1.8 in CECC group.^23^ In a meta-analysis including CABG and aortic valve and aortic root surgery, MiECC reduced the need of RBC transfusions by 45% compared to CECC.^24^ On the other hand, Ellam et al. reported no significant difference in need of RBC units in patients undergoing valve surgery in general.^25^

The need of a high number of RBC units has been associated with increased morbidity and mortality. In a study of 12,000 CABG patients, each RBC unit transfused was associated with a 77% increase in the risk of mortality,^26^ and in another study of cardiac surgery patients, RBC transfusion of at least 5 units was associated with an 8-fold increase in the odds of death.^16^ In a third study of cardiac surgery patients, each RBC unit transfused was associated with a 29% increase in crude risk of major infection.^27^ In our study, the 30-day mortality was only 0.8%, which is low compared to Bramer et al., who reported 7% 30-day mortality in mitral valve surgery patients.^28^ In our study, no significant difference in 30-day mortality was found between MiECC and CECC groups. However, mortality was low in both groups and thus, our study had low power to address differences in mortality. In a meta-analysis including 1619 patients (mainly CABG), the in-hospital mortality was 1.0% in MiECC group and 1.8% in CECC group (*p* = 0.23).^24^

The risk of NOAF is higher after mitral valve surgery than after other cardiac surgery. The volume load leads to left atrial (LA) enlargement, which predisposes to atrial fibrillation.^29^ After mitral valve surgery the reverse LA remodelling may induce reversal of electrophysiologic abnormalities, which increases the risk of atrial fibrillation.^30^ In our study, the incidence of NOAF was 41.8%, which is in line with previous studies. In a study of mitral valve surgery patients, the incidence of NOAF was 40.2%.^28^ In another study the incidence of NOAF was 28% in isolated mitral valve repair, 32% in isolated mitral valve replacement and 41% in combined mitral valve repair and CABG.^9^

Although the relatively high risk for NOAF after mitral valve surgery is well known and there are some data indicating that MiECC over CECC might be favorable as to the risk of NOAF, there are only few studies comparing the use of MiECC and CECC in mitral valve surgery and the results have been controversial. In the present study, we found no difference in the incidence of NOAF between MiECC and CECC groups in mitral valve surgery patients. In several previous studies representing various types of cardiac surgery, a lower incidence of NOAF has been reported after MiECC compared with CECC. In the study by Immer et al., the incidence of NOAF in CABG patients was significantly lower in MiECC group (11%) than in CECC group (39%).^22^ In another study of CABG patients comparing the occurrence of atrial fibrillation or atrial flutter, the incidence of NOAF was 25% in MiECC group and 36% in CECC group.^23^ However, a meta-analysis including 1619 patients (mainly CABG) showed no significant difference between MiECC and CECC groups (30% vs. 34%).^24^ This is in line with our current study as well as with the previous randomized trial in which 240 CABG patients were randomized to MiECC and CECC in which the incidence of NOAF was 35.0% in MiECC group and 35.8% in CECC group.^7^

There is a potential link between perfusion and RBC transfusion with NOAF, i.e. the inflammatory response caused by both extracorporeal perfusion as well as by blood products known to be one of the predisposing factors for NOAF. In a study population of over 5800 CABG patients with or without valve replacement, intensive care unit RBC transfusion increased the risk for NOAF with an odds ratio of 1.18 (95% CI 1.14–1.23) per unit transfused.^5^ In another study including CABG patients the odds of NOAF increased 61% with each increasing level of RBC transfusion.^4^ The likely explanation is that RBC transfusion modulates the inflammatory response to cardiac surgery by changing the concentrations of inflammatory mediators and augmenting the inflammatory response.^31^ Although the association between RBC and NOAF was statistically significant only in the unadjusted analysis, the finding is consistent with previous reports.

Perfusion time and aortic cross-clamp time in our study were approximately 15 min shorter in MiECC than in CECC group. Previous studies in patients with CABG have shown similar results, although the difference has been smaller than in our study.^32,33^ A study of patients with combined CABG and aortic valve replacement showed shorter perfusion and total operative time in the MiECC group but no difference was seen in aortic cross-clamp time.^34^ However, there are several studies (mainly focusing on CABG patients) in which no differences have been observed in perfusion and aortic cross-clamp time.^7,21,22,25,35,36^

We acknowledge some limitations. First, we did not have data on possible NOAF after discharge. Therefore, the cumulative incidence of NOAF (including follow-up) may be underestimated. However, the peak of NOAF takes place within the first 4 postoperative days.^9,37^ Secondly, different cardioplegia methods were used for MiECC and CECC groups. Calafiore-type warm blood cardioplegia is used routinely for MiECC to avoid excessive hemodilution and to achieve mandatory cardioplegia concentrate volume reduction, which is a conceptual choice for minimized perfusion circuit. In turn, Buckberg-type tepid blood cardioplegia is the routine method in our clinic for CECC. However, it is unlikely that the difference in the cardioplegia method would explain the findings. Falcoz et al. observed no statistical difference between the groups concerning NOAF in their prospective randomized study comparing tepid and warm blood cardioplegia.^38^ The present study was conducted retrospectively and that does set some inherent limitations for the interpretation of the results. Moreover, this was a single-center study and thus the results cannot be generalized to all mitral valve surgery patients.

In conclusion, in patients undergoing mitral valve surgery MiECC was associated with less need of RBC transfusions than CECC, particularly among patients under the age of 65 years. However, no significant differences in the incidence of NOAF were observed in relation to the method of perfusion. Use of RBC transfusions was associated with increased risk of NOAF significantly only in unadjusted analysis. Prospective randomized trials would be valuable for identifying the patient groups benefitting from use of MiECC over CECC in mitral valve surgery.
